# Toxicity of standing milkvetch infected with *Alternaria gansuense* in white mice

**DOI:** 10.3389/fvets.2024.1477970

**Published:** 2025-01-07

**Authors:** Bo Yang, Zhi Biao Nan, Yan Zhong Li

**Affiliations:** State Key Laboratory of Herbage Improvement and Grassland Agro-Ecosystems, College of Pastoral Agriculture Science and Technology, Engineering Research Center of Grassland Industry, Ministry of Education, Lanzhou University, Lanzhou, China

**Keywords:** diseased plant, feeding experiment, hematology, histology, serum enzyme, toxicity

## Abstract

**Introduction:**

Standing milkvetch (*Astragalus adsurgens*) is widely distributed in the wild in Eurasia and North America and has been bred for cultivated forage in China. Yellow stunt and root rot disease caused by *Alternaria gansuense* is the primary disease of standing milkvetch. *A. gansuense* promotes the production of swainsonine in the plant. This study aimed to determine the safety of standing milkvetch that is infected with *A. gansuense* as forage for animals.

**Methods:**

Two-week-old specific pathogen-free (SPF) male white mice were fed a commercial mouse feed (CMF), healthy plant feed (HPF) and diseased plant feed (DPF) for 3 or 6 weeks. We observed histological changes in the liver and kidney tissues of the mice and measured their daily feed intake, daily water intake, body weight, feed utilization, organ coefficients, and activities of serum enzymes.

**Results:**

The results showed that the daily feed intake of the mice that were fed DPF and HPF was significantly higher (*p* < 0.05) than those fed CMF at 3 and 6 weeks. The highest increase was observed in the daily water intake of the mice fed HPF (*p* < 0.05) followed by DPF and CMF. However, the mice fed DPF gained the least weight (*p* < 0.05). There was a significantly higher percentage of liver weight to body weight of the mice fed DPF (*p* < 0.05) than those fed HPF for 3 weeks and those fed CMF for 3 and 6 weeks. There were significantly higher levels of concentrations of alanine aminotransferase in the mice fed DPF and HPF than those fed CMF for 3 weeks (*p* < 0.05) and 6 weeks (*p* < 0.01). However, there was no significant difference in the mice fed HPF than those fed DPF. There were significantly higher of lactate dehydrogenase concentration (*p* < 0.001), while the blood urea nitrogen was lower in the mice fed DPF than those fed HPF and CMF at 3 weeks. There was a significantly higher percentage of numbers of lymphocytes in the blood of the mice fed DPF (*p* < 0.05) than those fed HPF, but the percentages of monocytes and eosinophils were significantly lower. Comparatively, there were more apparent pathological changes in the liver and kidney tissues of the mice fed with DPF than in those fed with HPF.

**Discussion:**

These findings indicate that standing milkvetch was toxic to white mice, and infection with *A. gansuense* increased its toxicity. Therefore, we conclude that standing milkvetch plants infected by *A. gansuense* must never be used as animal feed under any circumstances. Additionally, the amount of healthy standing milkvetch fed to animals should be appropriate, avoiding long-term or excessive feeding.

## 1 Introduction

Standing milkvetch (*Astragalus adsurgens*), which is widely planted in northern China, is a palatable and nutritious forage for livestock and poultry (1, 2). It grows rapidly and is characterized by tolerance to soil with low nutrients, wide adaptability, and strong resistance; therefore, it plays an important role in the restoration of degraded ecosystems (3, 4).

Fungal diseases are a major limiting factor of standing milkvetch in pastures (5). Yellow stunt and root rot (YSRR) of standing milkvetch is the most important fungal disease of standing milkvetch, and it was first reported in Huanxian, Gansu. The disease was later reported in Ningxia, Shaanxi, Inner Mongolia, Yunnan, and Beijing, which are the primary areas of standing milkvetch production. The incidence of the disease can be as high as 82.6%, with a disease index of 53.7 (6). YSRR is a systemic disease that causes various symptoms, including yellowing, crumpling or even defoliation, plant dwarfing, dark brown stalks in the late stages of the disease, and the rotting of stem bases, rhizomes, and main roots, as well as death, which seriously exacerbates the degradation of grasslands (7).

The causative pathogen of YSRR was identified as a new species in 2007 based on its morphological characteristics and named *Embellisia astragali* (8). Later, *E. astragali* was renamed *Alternaria gansuense* based on its molecular and morphological characteristics (9).

The pathogen *A. gansuense* is closely related to the locoweed endophyte *A.* sect. *Undifilum* spp., which produces poisonous swainsonine and causes locoism in livestock (9, 10). Previous studies have shown that both diseased plants infected by *A. gansuense* and healthy uninfected plants contain swainsonine, but its content is higher in diseased plants than in healthy ones (11).

Higher contents of swainsonine of locoweed that reach or exceed 0.001% can cause livestock poisoning with continuous feeding. However, a low content of swainsonine owing to a low intake of locoweed can lead to weight loss without causing livestock poisoning (12). The concentration of alkaline phosphatase (ALP) and aspartate aminotransferase (AST) increased in the serum of rats fed solely with the endophyte or locoweed, while the concentration of *α*-mannosidase (AMA) decreased (11). In addition, vacuolization appeared on the pancreatic, renal, and hepatic tissues of the rats that were fed locoweed (13). Swainsonine is one of the key toxins produced by locoweed (14–18) and causes enormous stockbreeding losses in China and the USA (19). A previous study revealed that locoweed plants free of endophytes were non-toxic (20). However, it is not clear whether healthy standing milkvetch and plants infected with *A. gansuense* can cause animal poisoning as locoweed does. Therefore, this study aimed to assess whether healthy standing milkvetch and plants infected with *A. gansuense* are safe for animals.

## 2 Materials and methods

### 2.1 Animals and feed

A total of 48 two-week-old specific pathogen-free (SPF) male white mice (*Mus musculus*) of the BABL/C variety, weighing 18–20 g, were obtained from the Animal Experimental Center, Lanzhou University, China, for the study. The experimental scheme was approved by the Lanzhou University College of Grassland Agricultural Science and Technology Ethics Committee.

Healthy and diseased standing milkvetch plants were collected from Huanxian County, Gansu Province, China. The diseased plants (infected with *A. gansuense*) had brown stems, while the healthy plants were free from any symptoms. The plants were oven-dried at 80°C for 24 h and then ground in a mill (FZ102, Yongguangming, China) using a 1-mm diameter screen. Commercial mouse feed (CMF) (Keao Xieli, China) was also ground with the same mill and separated into three parts. The diseased and healthy foliage powder was added to the first and second parts of the CMF powder at a weight of 20%, respectively, and no foliage powder was added to the third part. Each part was mixed thoroughly, and a fodder machine (KL120-200, Xinghui, China) was used to create a 40-mm long cylinder that was 12 mm in diameter. The three mouse feeds were then added to 25% water of the total weight and dried at 80°C for 12 h, respectively. The feeds that contained healthy and diseased standing milkvetch powder were named healthy plant feed (HPF) and diseased plant feed (DPF), respectively. The contents of crude protein, crude fat, crude fiber, moisture, ash, calcium, and phosphorus of the three mouse feeds were then measured (21).

### 2.2 Mice feeding with the three feed types

The study included 3-week and 6-week experiments to assay the serum enzymes and another 6-week experiment for the hematology assay. In each experiment, eight male mice were fed CMF, HPF, and DPF. For each experiment, two male mice were kept in a 45 cm × 30 cm × 28 cm plastic cage. The cages were randomly arranged in a block in a feeding instrument equipped with an aeration system where many pipes were individually connected to each cage (Fengshi, IVC-II, China). The instrument was kept at a room temperature of 18–22°C under a 12 h light/12 h dark photo period and 30–60% relative humidity. The room was disinfected with 40% formalin and UV light for 24 h before the experiment. The feeds were placed onto the net atop the cages, and distilled ion-ex water was supplied in the drinking bottles for the mice.

### 2.3 Measurement indicators during the feeding period

The daily feed intake and body weight of the two mice in cages were measured using an electronic balance (CP324S, Odolish, China), and the daily water intake was measured using a graduated flask at 9:00 a.m. every day. The eating, drinking, movement, and sleep of the mice were observed daily during the feeding period to determine any signs of poisoning. The daily feed and water intake were calculated as follows:

Daily feed intake = The feed weight fed the previous day - Remaining feed weight the next day.

Daily water intake = The water amount fed the previous day - Remaining water amount the next day.

### 2.4 Measurement indicators after the mice had been euthanized

#### 2.4.1 Tissue weight and histology

The mice were euthanized at weeks 3 and 6, and the heart, liver, pancreas, spleen, and kidney tissues were excised and weighed. The tissues were subsequently observed to determine any pathogenic signs and then stored in glass jars in 10% formalin. A histological analysis was performed by embedding the tissues in paraffin, followed by sectioning, staining with hematoxylin and eosin, and observation under a microscope (Olympus, NC300-1, Tokyo, Japan).

#### 2.4.2 Serum enzymes

Blood samples were obtained from the eyes of mice during both the 3-week and 6-week experiments (conducted for serum enzyme assay) and were allowed to incubate stationary for 12 h. The serum was separated by centrifugation (XKA-2200, Xiangyi, China). The activities of serum ALP, alanine aminotransferase (ALT), AST, and lactate dehydrogenase (LDH) and the content of blood urea nitrogen (BUN) were immediately assayed with an automatic biochemical analytical instrument (XL-300, Erba, Mannheim, Germany). *α*-Mannosidase was assayed using a mouse α-mannosidase ELISA kit (USCN Life Science, USA) and an auto ELISA detector (RT-6100, Rayto, USA) according to the manufacturer’s instructions.

#### 2.4.3 Hematology

Blood samples were collected from the eyes of mice during the 6-week experiment (conducted for hematology assay) and were transferred into tubes containing malondialdehyde (MDA). The hematology analysis was conducted using an automatic animal blood analysis instrument (Hemavet 950, Drew Scientific, Plantation, FL, USA).

### 2.5 Statistical analysis

The feed and water intake data from the two mice in each cage were averaged. The feed utilization efficiency (FUE) was calculated using the weight gained and total intake (22) as follows:


$$FUE=\frac{\mathrm{The}\;\mathrm{final}\;\mathrm{body}\;\mathrm{weight}–\mathrm{The}\;\mathrm{initial}\;\mathrm{body}\;\mathrm{weight}}{\mathrm{The}\;\mathrm{total}\;\mathrm{food}\;\mathrm{intake}}\times 100\%$$

In the formula, “the total food intake” represents the total amount of food consumed by the two mice in each cage, and the weight gain represents the combined total of the two mice in each cage.

The organ coefficient (OC) for each tissue was calculated using the weight of each tissue and body weight (23) as follows:


$$OC=\frac{\mathrm{Each}\;\mathrm{tissue}\;\mathrm{weight}}{\mathrm{Body}\;\mathrm{weight}}\times 100\%$$

The means were subjected to a one-way ANOVA using SPSS 11.5.0 (SPSS Inc., Chicago, IL, USA) and compared using Duncan’s new multiple range test.

## 3 Results

### 3.1 Nutrient components of the feeds

The contents of crude protein and ash of the healthy and diseased plant feeds were slightly higher than those of the CMF. There were much higher contents of crude fiber in the two plant feeds, but their contents of crude fat and non-nutrient extracts were slightly lower than those in the CMF. However, there were no significant differences between the two types of plant feed (Table 1).

**Table 1:** Nutrient level of three mouse feed (%).

Nutrient	Commercial feed (CMF)	Healthy plant feed (HPF)	Diseased plant feed (DPF)
Crude protein	17.92	18.0	18.5
Crude fat	5.00	4.24	4.04
Crude fiber	3.52	9.52	9.59
Non-nutrient extract	48.37	45.18	47.54
Ash	5.48	6.32	6.03

### 3.2 Feed and water intake, gained weight, and feed utilization

There was a significantly higher mean daily intake for the DPF and HPF than for the CMF during the 3-week (*p* < 0.01) and 6-week (*p* < 0.01) experiments. However, there were no significant differences between the DPF and HPF.

The mice fed DPF had the lowest mean daily water intake, while those fed the HPF had the highest in the 3-week (*p* < 0.05) and 6-week (*p* < 0.05) experiments.

The least amount of total weight gained by the mice fed the DPF occurred in weeks 3 and 6 (*p* < 0.05), but the highest amount occurred in those fed the CMF for 3 weeks and those fed the HPF for 6 weeks.

The utilization efficiency was the lowest for the DPF in all the experiments (*p* < 0.05), but the highest for the CMF in the 3-week experiment and for the HPF in the 6-week experiment (Table 2).

**Table 2:** Feed and water intake, gained weight and feed utilization efficiency of white mice fed with the feed contained 20% diseased standing milkvetch foliage infected by *A. gansuense* compared with fed with feed contained 20% healthy standing milkvetch foliage and a commercial feed for 3 and 6 wks.

Items	Feeding period (wks)					
	3			6		
	Commercial feed (CMF)	Healthy plant feed (HPF)	Diseased plant feed (DPF)	Commercial feed (CMF)	Healthy plant feed (HPF)	Diseased plant feed (DPF)
Mean daily feed intake (g/ d·mouse)	3.42 ± 0.15b^1^	4.26 ± 0.12a	4.32 ± 0.12a^**^	3.51 ± 0.15b^2^	4.13 ± 0.14a^**^	4.16 ± 0.05a
Mean daily water intake (ml/d·mouse)	5.02 ± 0.06b	5.8 ± 0.14a^*^	5.37 ± 0.24ab	5.09 ± 0.04b	5.73 ± 0.20a^*^	5.52 ± 0.20ab
Total gained weight (g/ d·mouse)	0.41 ± 0.52a^*^	0.07 ± 0.31b	−0.2 ± 0.25c	1.57 ± 0.95b	2.38 ± 0.47a*	0.84 ± 0.46c
Feed utilization efficiency (%)	0.54 ± 0.74a^*^	0.05 ± 0.34b	−0.24 ± 0.29c	1.02 ± 0.32a	1.3 ± 0.14a^*^	0.48 ± 0.14b

^1^Data in the same row were compared using Duncan’s new multiple range test (*P < 0.05, **P < 0.01, ***P < 0.001).

### 3.3 Organ coefficient

**Figure 1:**
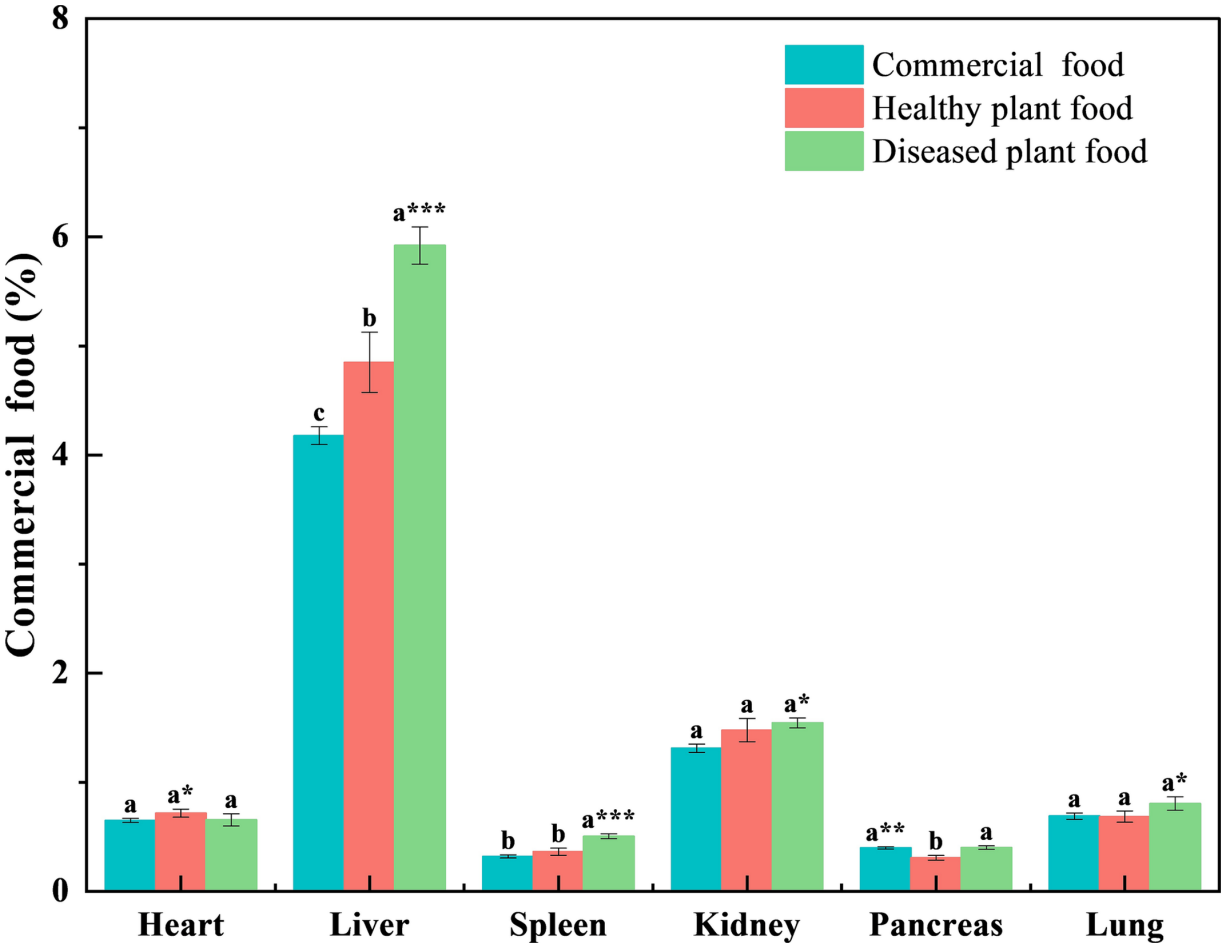
Organ coefficient of white mice fed with feed that contained 20% of standing milkvetch foliage infected with *Alternaria gansuense* compared to those fed with feed that contained 20% of healthy standing milkvetch foliage and a commercial feed for 3 weeks. Data (mean ± SE) within the same organ were compared using Duncan’s new multiple range test. *p < 0.05, **p < 0.01, and ***p < 0.001. Bars topped by the same lowercase letter do not differ significantly between treatments.

**Figure 2:**
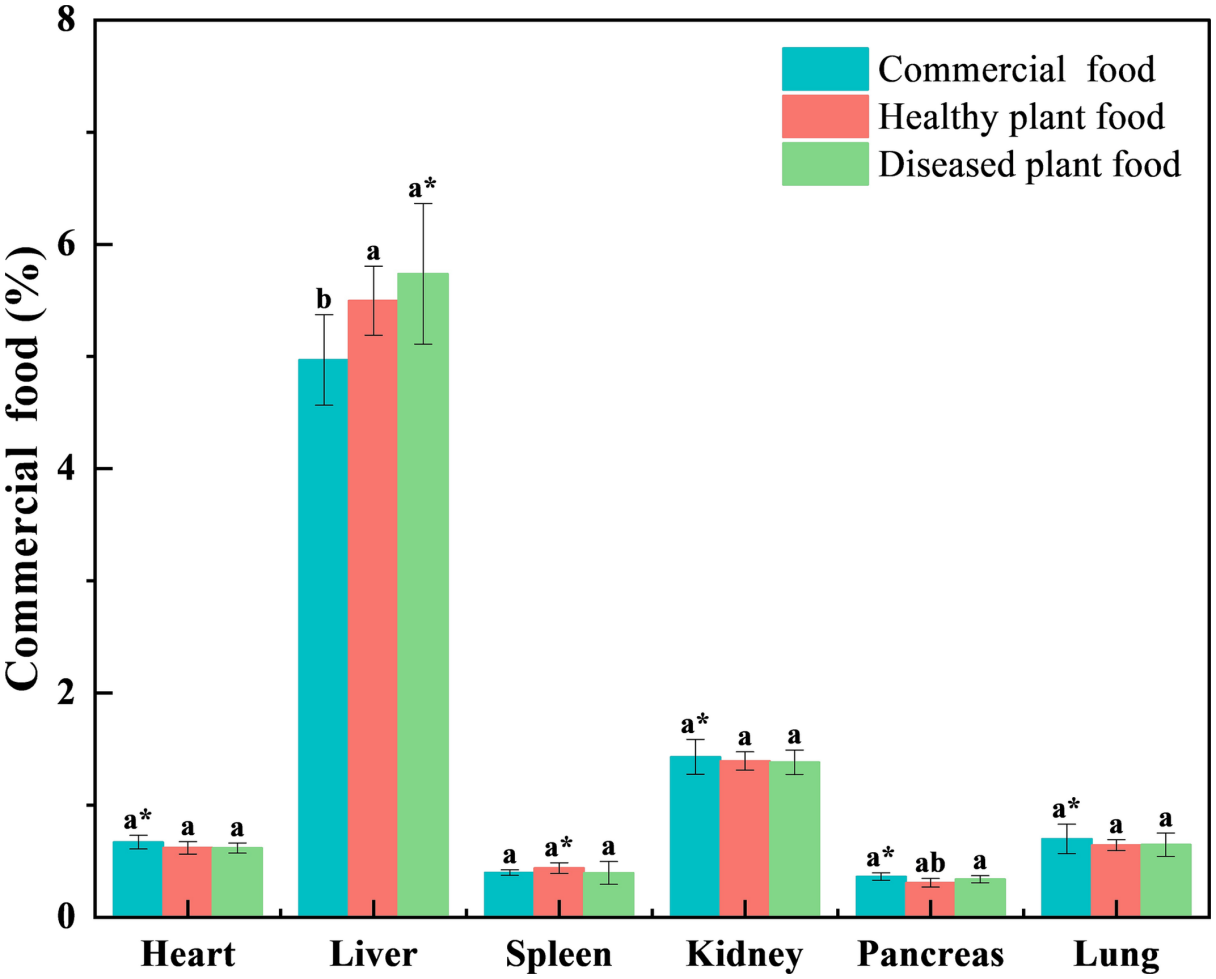
Organ coefficient of white mice fed with feed that contained 20% of standing milkvetch foliage infected with *Alternaria gansuense* compared to those fed with feed that contained 20% of healthy standing milkvetch foliage and a commercial feed for 6 weeks. Data (mean ± SE) within the same organ were compared using Duncan’s new multiple range test. *p < 0.05, **p < 0.01, and ***p < 0.001. Bars topped by the same lowercase letter do not differ significantly between treatments.

Liver coefficients of the mice fed the DPF were significantly greater than those of mice fed the CMF for 3 weeks (*p* < 0.001) and 6 weeks (*p* < 0.05) (Figures 1, 2) and mice fed the HPF for 3 weeks (Figure 1). There was a significantly higher spleen coefficient in the mice fed the DPF (*p* < 0.01) than in those fed the CMF and HPF for 3 weeks (Figure 2). Furthermore, there was a significantly higher pancreas coefficient in the mice fed the DPF (*p* < 0.05) than in those fed the HPF for 3 weeks, but it was not significantly different (*p* > 0.05) from those fed the HPF for 6 weeks. There was no difference in the pancreas coefficient between the mice fed the DPF and those fed the CMF (Figures 1, 2).

### 3.4 Serum enzyme activities

At 3 and 6 weeks of feeding, the ALT concentrations in the serum of mice were as follows: 36.37 ± 9.30 and 24.49 ± 2.68 U/L for the DPF group, 34.03 ± 9.69 and 29.41 ± 3.74 U/L for the HPF group, and 11.26 ± 0.91 and 14.83 ± 1.88 U/L for the CMF group, respectively. There were significantly higher concentrations of ALT in the mice fed DPF and HPF than in those fed CMF for 3 weeks (*p* < 0.05) and 6 weeks (*p* < 0.01). However, mice fed the HPF did not differ significantly from those fed the DPF.

At 3 and 6 weeks of feeding, the BUN concentrations were as follows: 2.15 ± 0.15 and 1.43 ± 0.30 mmol/L in the DPF group, 1.71 ± 0.16 and 1.79 ± 0.14 mmol/L in the HPF group, and 2.36 ± 0.18 and 1.44 ± 0.13 mmol/L in the CMF group, respectively. The BUN concentration in the mice fed the DPF was between the BUN concentrations of those fed the HPF and CMF, while the mice fed the HPF had a significantly lower (*p* < 0.05) concentration of BUN than those fed CMF for 3 weeks. There was no significant difference in the concentration of BUN between the groups at 6 weeks.

At 3 and 6 weeks of feeding, the LDH concentrations were as follows: 269.07 ± 14.09 and 166.30 ± 16.31 U/L in the DPF group, 154.50 ± 12.66 and 218.40 ± 27.59 U/L in the HPF group, and 210.04 ± 12.49 and 223.14 ± 14.48 U/L in the CMF group, respectively. The mice fed DPF had significantly higher levels of LDH concentration than those fed HPF and CMF, and the mice fed HPF had significantly lower levels of LDH concentration than those fed CMF for 3 weeks. There was no significant difference in the concentration of LDH between the groups at 6 weeks.

At 3 weeks of feeding, the AMA, AST, and ALP concentrations were as follows: 2.20 ± 0.05, 89.01 ± 8.56, and 53.15 ± 5.55 U/L in the DPF group, 2.03 ± 0.05, 73.01 ± 12.45, and 44.74 ± 7.48 U/L in the HPF group, and 2.23 ± 0.11, 62.99 ± 6.75, and 44.54 ± 7.73 in the CMF group, respectively. At 6 weeks of feeding, the AMA, AST, and ALP concentrations were as follows: 1.97 ± 0.07, 58.83 ± 10.98, and 55.41 ± 3.56 U/L in the DPF group, 2.26 ± 0.16, 70.01 ± 5.57, and 46.86 ± 8.96 U/L in the HPF group, and 2.08 ± 0.05, 62.70 ± 7.79, and 50.54 ± 6.63 in the CMF group, respectively. There were no significant differences in the AMA, AST, and ALP concentrations between the groups at 3 and 6 weeks (Table 3).

**Table 3:** Serum enzyme activities of mice fed with three feed for 3 and 6 wks.

Enzymes^1^	Feeding period (wks)					
	3			6		
	Commercial feed (CMF)	Healthy plant feed (HPF)	Diseased plant feed (DPF)	Commercial feed (CMF)	Healthy plant feed (HPF)	Diseased plant feed (DPF)
AMA (U/L)	2.23 ± 0.11a^2^	2.03 ± 0.05a	2.20 ± 0.05a	2.08 ± 0.05a	2.26 ± 0.16a	1.97 ± 0.07a
ALT (U/L)	11.26 ± 0.91b	34.03 ± 9.69a	36.37 ± 9.30a^*^	14.83 ± 1.88b	29.41 ± 3.74a^**^	24.49 ± 2.68a
AST (U/L)	62.99 ± 6.75a	73.01 ± 12.45a	89.01 ± 8.56a	62.70 ± 7.79a	70.01 ± 5.57a	58.83 ± 10.98a
ALP (U/L)	44.54 ± 7.73a	44.74 ± 7.48a	53.15 ± 5.55a	50.54 ± 6.63a	46.86 ± 8.96a	55.41 ± 3.56a
BUN (mmol/L)	2.36 ± 0.18a^*^	1.71 ± 0.16b	2.15 ± 0.15ab	1.44 ± 0.13a	1.79 ± 0.14a	1.43 ± 0.30a
LDH (U/L)	210.04 ± 12.49b	154.50 ± 12.62c	269.07 ± 14.09a^***^	223.14 ± 14.48a	218.40 ± 27.59a	166.30 ± 16.31a

^1^AMA, α-mannosidase; ALP, alkaline phosphatase; ALT, alanine aminotransferase; AST, aspartate aminotransferase; LDH, lactate dehydrogenase; BUN, blood urea nitrogen. ^2^Data in the same row were compared using Duncan’s new multiple range test (*P < 0.05, **P < 0.01, ***P < 0.001).

### 3.5 Hematology

There were significantly higher numbers of eosinophils in the blood (*p* < 0.05) in the mice fed the HPF than in those fed the CMF. However, the levels were similar to those fed the DPF. There was a significantly higher percentage of neutrophils in the blood of mice fed the DPF and HPF than in those fed the CMF, and there was no significant difference between the DPF and HPF (*p* < 0.001). There was a significantly higher percentage of lymphocytes in the blood of mice fed the DPF and CMF than in the mice fed the HPF, and there was no significant difference between the DPF and CMF (*p* < 0.001). The percentage of monocytes in the blood of mice fed the HPF was significantly higher than that of mice fed the DPF (*p* < 0.01). There was a significantly higher percentage of eosinophils in the blood of mice fed the HPF than in those fed the DPF and CMF, and there was no significant difference between the DPF and the CMF (*p* < 0.05). There was no significant difference in the numbers of white blood cells, neutrophils, lymphocytes, monocytes, basophils, red blood cells, and platelets in the blood of mice fed the DPF, HPF, and CMF (Table 4).

**Table 4:** Hematology of mice fed three feed for 6-wks.

Items	Commercial feed (CMF)	Healthy plant feed (HPF)	Diseased plant feed (DPF)	Items	Commercial feed (CMF)	Healthy plant feed (HPF)	Diseased plant feed (DPF)
White blood cell (WBC, K/uL)	3.47 ± 0.66a^1^	3.31 ± 0.10a	3.45 ± 0.20a	Basophil (BA, %)	0.52 ± 0.19a	1.09 ± 0.30a	0.44 ± 0.14a
Neutrophil (NE, K/uL)	0.88 ± 0.20a	1.25 ± 0.06a	1.15 ± 0.15a	Red blood cell (RBC, M/uL)	9.04 ± 1.04a	10.20 ± 0.18a	9.60 ± 0.37a
Lymphocyte (LY, K/uL)	2.19 ± 0.40a	1.48 ± 0.10a	1.96 ± 0.08a	Hemoglolin (Hb, g/dL)	12.49 ± 1.55a	14.30 ± 0.25a	13.54 ± 0.61a
Monocyte (MO, K/uL)	0.28 ± 0.08a	0.41 ± 0.07a	0.270.03 ± a	Hematocrit, Packed cell volume (HCT, %)	30.15 ± 3.58a	33.08 ± 0.57a	30.83 ± 1.30a
Eosinophil (EO, K/uL)	0.03 ± 001b	0.10 ± 0.02a^*^	0.05 ± 0.01ab	Mean corpuscular volume (MCV, fL)	30.68 ± 2.35a	32.43 ± 0.23a	32.08 ± 0.25a
Basophil (BA, K/uL)	0.02 ± 0.01a	0.04 ± 0.01a	0.02 ± 0.01a	Mean corpuscular hemoglobin (MCH, Pg)	14.19 ± 1.11a	14.05 ± 0.11a	14.08 ± 0.16a
Neutrophil (NE, %)	22.43 ± 2.58b	37.61 ± 1.58a^***^	32.50 ± 2.90a	Mean corpuscular hemoglobin concentration (MCHC, g/dL)	43.01 ± 3.68a	43.24 ± 0.29a	43.88 ± 0.19a
Lymphocyte (LY, %)	65.58a ± 2.56a^***^	44.79 ± 2.73b	57.95 ± 3.58a	Red blood cell volume distribution width (RDW, %)	16.44 ± 0.33a	16.59 ± 0.11a	16.18 ± 0.16a
Monocyte (MO, %)	10.03 ± 1.1.ab	13.54 ± 1.72a^*^	7.67 ± 0.63b	Platelet (PL, K/uL)	534.38 ± 41.37a	517.25 ± 47.82a	539.38 ± 35.02a
Eosinophil (EO, %)	1.17 ± 0.43b	2.98 ± 0.69a^*^	1.45 ± 0.33b	Meam platelet volume (MPV, fL)	5.73 ± 0.07a	5.88 ± 0.11a	5.83 ± 0.05a

^1^Data in the same row were compared using Duncan’s new multiple range test (*P < 0.05, **P < 0.01, ***P < 0.001).

### 3.6 Clinical signs

No signs of poisoning were observed during the entire experiment.

### 3.7 Histology

The liver tissues of the mice fed the DPF and HPF were apparently larger than those fed the CMF, and some were dropsical with blood clots. There were no obvious changes in the organs.

**Figure 3:**
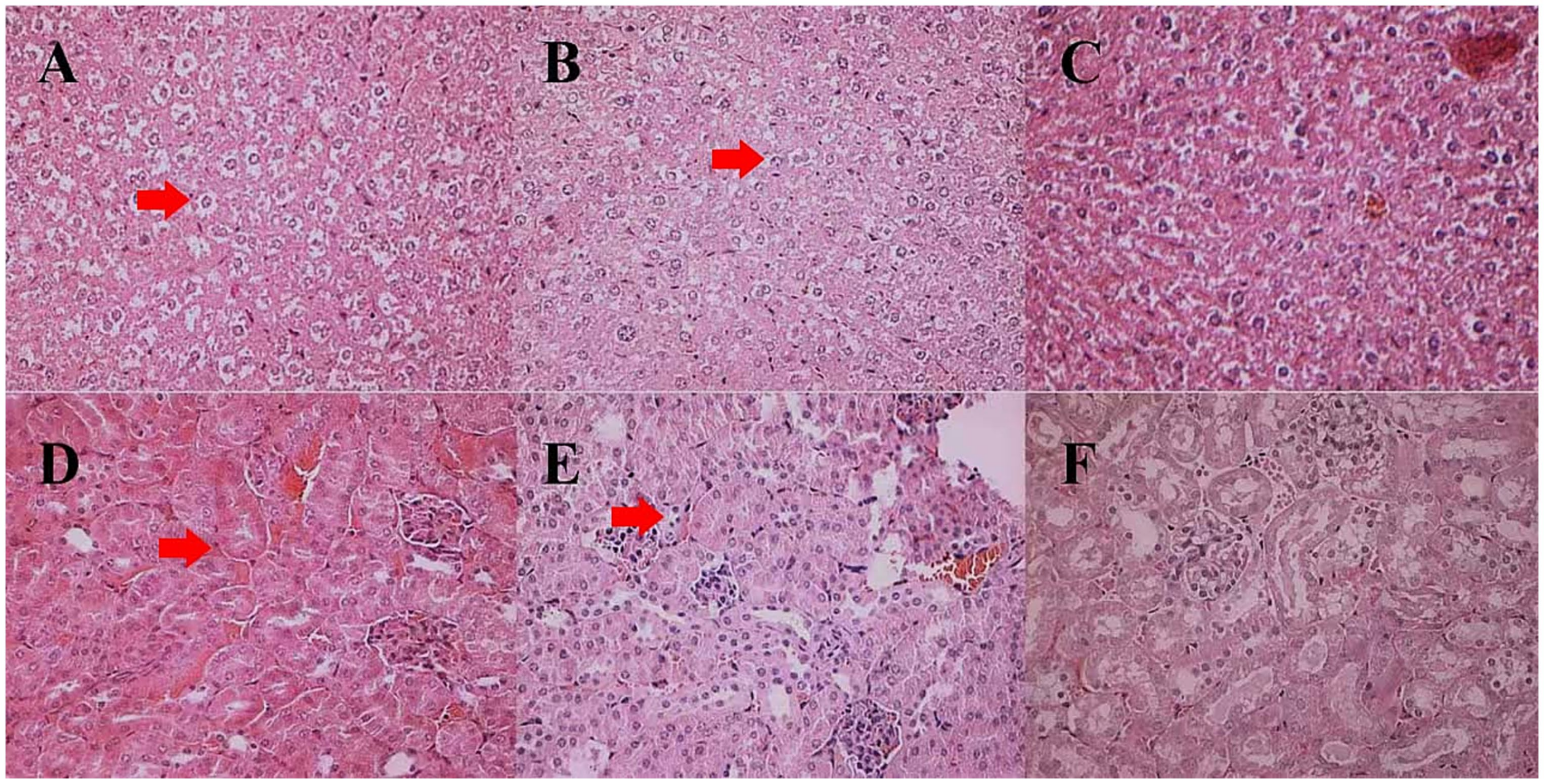
Histological changes in the liver and kidney tissues of white mice fed with feed that contained 20% of standing milkvetch foliage infected with *Alternaria gansuense* compared to those fed with feed that contained healthy standing milkvetch foliage and a commercial feed for 6 weeks. **(A–C)** Liver: healthy plant feed (HPF), diseased plant feed (DPF), and commercial feed (CMF), respectively. **(D–F)** Kidney: healthy plant feed (HPF), diseased plant feed (DPF), and commercial feed (CMF), respectively. In **(A,B)** the locations pointed to by red arrows show binucleated hepatocytes and vacuolation. In **(D,E)** the locations pointed to by red arrows show hypertrophy, hyperplasia, and proliferation of mesangial cells in the glomeruli.

Compared to the tissue sections of mice fed the CMF (Figures 3C,F), pathological changes appeared more severe in the liver and kidney tissues of the mice fed the DPF and HPF.

The liver antra of the mice fed the HPF and DPF became narrower, with enlarged and swollen liver cells. Some cells at the edge of the liver lobule were necrotic, with double nucleoli or light-stained plasma. Cells in the center of the liver lobule were larger, with double nucleoli and large or small vacuoles (Figures 3A,B). Hypertrophy proliferation and hyperplasia were observed in the glomerular mesangial cells. Renal tubule epithelial cells were granularly degenerated, and the renal tubulointerstitial cells were dilated and congestive (Figures 3D,E).

## 4 Discussion

Standing milkvetch is a nutritive fodder that has been planted for a long time in China, although it is listed in the noxious plant catalog in the United States and Canada (10). This study found that healthy standing milkvetch plants were noxious to mice, as the liver coefficient and concentration of ALT were higher in the mice fed healthy standing milkvetch plants than those fed the CMF. Additionally, the viscera of the mice fed the HPF exhibited pathological changes. Many locoweeds (*Oxytropis* and *Astragalus* plants) are poisonous to livestock (13, 24). Their toxins are acknowledged to be selenium (25), nitrogenous compounds (26), and swainsonine (27).

In recent years, the toxicity of locoweed has primarily been attributed to swainsonine (28, 29). A series of obvious signs of poisoning were reported in chickens, which demonstrated that standing milkvetch is toxic to poultry (26). However, there were no evident signs of poisoning in rabbits and sheep (30). In these previous studies, hematology, activities of LDH, ALP, and ALT, and the content of hemoglobin were similar to those of the controls, although some pathological changes were observed on the viscera. Generally, the content of 3-nitropropionic acid (3-NPA) is lower in the stems, leaves, and flowers of standing milkvetch (31), and its digestion by ruminants transforms it into an innocuous compound. Therefore, some authors have considered standing milkvetch to be safe for animals and unlikely to induce toxicosis in livestock (26). However, this study found enlarged liver tissues in the mice fed the HPF. Additionally, it showed that healthy standing milkvetch plants were noxious to mice, as the liver coefficient and ALT concentration were higher in the mice fed healthy standing milkvetch plants than in those fed the CMF. Furthermore, the viscera of the mice fed the HPF exhibited pathological changes. The activity of alanine aminotransferase in the 3 week and 6 week experiments and the contents of BUN in the 3-week experiment, and the neutrophils, eosinophils, and lymphocytes in the 6-week experiment were abnormal compared to the mice fed the CMF. The decrease in BUN may correlate with liver damage (32). Eosinophils are often associated with allergies, while the increase in lymphocytes occurs because of inflammation (33). In this study, the hematology reports of the mice were compared to the reference ranges for the species (34), and no abnormalities were detected. Thus, standing milkvetch forage is harmful to animals, although no clear signs of poisoning were observed.

The DPF was more toxic than the HPF, as shown by the lower weight gain and feed utilization efficiency, the higher liver coefficient, and more severe pathological changes in most of the tissues of the mice fed DPF. Previous studies have shown that swainsonine is the primary toxic substance in standing milkvetch plants (35), and its content is higher in diseased plants than in healthy ones. *A. gansuense* can elevate the production of swainsonine (11). This is consistent with our research findings, and therefore, we conclude that swainsonine is the primary substance responsible for poisoning in mice. Swainsonine is an indolizidine alkaloid that is structurally similar to mannose and has a strong inhibitory effect on *α*-mannosidase. This inhibition disrupts the processing of N-glycans, as α-mannosidase is a key enzyme in the processing of N-glycan chains. By inhibiting α-mannosidase, swainsonine prevents the processing of glycoproteins, leading to the accumulation of unprocessed sugar chains at the mannose position. The accumulation of these sugar chains interferes with the normal function of glycoproteins, including their roles on the cell surface (35). The action mechanism of swainsonine leads to a series of biological effects, including the accumulation of complex oligosaccharides within cells, particularly in lysosomes, which can cause cellular vacuolation, especially in neurons (36). Additionally, swainsonine poisoning can affect the immune system and result in damage to the liver and kidneys (37). This substance can cause poisoning in the vast majority of animals, such as cattle, sheep, horses, and mice (38).

Research has demonstrated that endophytic fungi coexist symbiotically with plants in the genus *Oxytropis*, enabling them to produce swainsonine. If the endophytic fungi are removed from these plants, they cease to produce swainsonine, resulting in plants that are no longer toxic (39). Are endophytic fungi present in standing milkvetch plants? If present, would removing the endophytic fungi from the plant make it non-toxic? These questions will be the focus of future research.

In summary, our research results indicate that standing milkvetch is toxic to mice and that its toxicity is exacerbated upon infection by *A. gansuense*. Therefore, we conclude that standing milkvetch plants infected by *A. gansuense* must never be used as animal feed under any circumstances. Additionally, the amount of healthy standing milkvetch fed to animals should be appropriate, avoiding long-term or excessive feeding. The limitation of this study resides in the fact that the principal consumers of this feed are ruminant animals, while the toxicity of the feed was evaluated using white mice in this study. Future research ought to center on ruminant animals.

## Data Availability

The datasets presented in this study can be found in online repositories. The names of the repository/repositories and accession number(s) can be found in the article/supplementary material.
